# Reflections on ethical challenges encountered in Indigenous health research using archival records

**DOI:** 10.3402/ijch.v75.32592

**Published:** 2016-06-27

**Authors:** Sylvia Abonyi, Paul Hackett, Roland Dyck

**Affiliations:** 1 SPHERU/Department of Community Health and Epidemiology, University of Saskatchewan, Saskatoon, SK, Canada. Email: sya277@mail.usask.ca; 2 SPHERU/Department of Geography and Planning, University of Saskatchewan, Saskatoon, SK, Canada; 3 Canadian Center for Health and Safety in Agriculture, Department of Medicine, College of Medicine, University of Saskatchewan, Saskatoon, SK, Canada

The dataset on which our paper is based comes from residential school students’ health examination records that form a small part of the Department of Indian Affairs’ Record Group 10 Collection (RG 10) (1). RG 10 is a vast and diverse collection that documents Canada's oft-troubled relationship with First Nations people. RG 10 is publicly available and held on microfilm in universities and other data repositories across Canada. While there is no way of knowing exactly what these records contain until they are closely scrutinized, these types of archival records are extremely important for documenting Canadian history. For example, analysis of RG 10 and related record groups has revealed appalling conditions and abuses that occurred in the residential school system (2,3).

As we looked closely at the health examination records used in our study, their contents challenged us to view historical research through two contemporary lenses: the Canadian principles for ethical research with Indigenous peoples that govern practices largely around the collection of new data (4); and the activities and outcomes of our national Truth and Reconciliation Commission (TRC) and its offshoot, the new National Centre for Truth and Reconciliation (NCTR), whose mandate includes truth-telling about the legacy of Indian residential schools (5,6).

The records we consulted date from the early- to mid-twentieth century, a time when Canadian Indigenous children were required to attend residential schools. Records were collected during routine health examinations carried out at school entry to assess children's overall health status and isolate those with infectious conditions such as tuberculosis. There is no evidence that these health examinations were carried out with the knowledge or consent of parents. These records include detailed, sensitive and *individually identifiable* material, including children's and parents’ names, home communities, educational background and health status. The records cover the period 1919–1953, therefore many of these children and their descendants are still alive today.

Archival material located in the public domain is not subject to any conditions or restrictions and is available to anyone with the wherewithal to wade through voluminous handwritten or typed material in files or on microfilm. University-based researchers using these data are under no obligation to have their research protocol reviewed or approved by a research ethics board. Nevertheless, our team appreciated the significance of the ethical challenges surrounding the use of these data and developed a strategy to work through them. That strategy is described here, and we hope it sparks conversations that lead to the development of new processes surrounding the use of historical data such as these.

First, we anonymized the data by removing children's names and other identifiers such as information on parents and communities of origin. Data were aggregated into clusters identified only by age, sex and location of the residential school. Despite anonymization and aggregation of data, the nature of the inquiry meant that we were nonetheless reporting on the lives and experiences of the First Nations people. It was therefore appropriate that we consulted with Indigenous partners and organizations as the work unfolded. Indigenous colleagues and organizations provided consultation throughout the research process. We are immensely grateful to those who read drafts of the paper or met with us to work through the concerns we have outlined here. Our preliminary findings were presented at several conferences, where we had more opportunities for discussion with a diverse audience that included Indigenous and non-Indigenous researchers and community members. We also benefited from the comments and suggestions of anonymous peer reviewers, who not only helped us strengthen the analysis but also contributed to the ethical dialogue.

Finally, it is important to note that this research took place as the TRC was travelling the country, gathering the stories of residential school survivors and their descendants. Among the Truth and Reconciliation Commission's 94 calls to action is the recommendation that Library and Archives Canada “ensure that its record holdings related to residential schools are accessible to the public” (7, p. 332). Overwhelmingly, our Indigenous colleagues affirm that the data from the health examinations tells an important part of the residential school story and that they should be used for this type of scholarly research, despite the circumstances under which they were collected. Notably, the records from which we were able to conduct this analysis are in the same RG10 file that houses a letter from Duncan Campbell Scott, the Deputy Superintendent General, in which he dismisses claims from children that they were hungry in school, arguing that, “Ninety-nine percent of the Indian children at these schools are too fat” (8). As important as it is to reveal these stories, our Indigenous colleagues expressed concern that detailed, identifiable information on children's health exams is available in the public domain. We share this concern and urge the research community to join the NCTR's efforts to put in place a process for the use of these data in ways that tell the truth without committing further harm.

Our consultation process does not end with publication. We see this editorial as part of our call for ongoing discussions about the importance of adapting principles, processes or approaches for ethical research involving the analysis of historical records about Indigenous peoples in Canada. At minimum, we assert that the accessibility of records of the residential school entrance exams, in particular in a form that reveals detailed personal and health information about named children, be carefully managed to ensure they are used in research in a manner consistent with contemporary Indigenous health research ethics guidelines in Canada (4). We are exploring opportunities for Indigenous stewardship of the electronic dataset we created from our transcription of the records. In this we are guided by the words of the TRC commissioners that ask all Canadians to commit to the establishment of new and respectful relationships between Aboriginal and non-Aboriginal Canadians so that we may “restore what must be restored, repair what must be repaired, and return what must be returned” (9, p. 1).

*Sylvia Abonyi* SPHERU/Department of Community Health and Epidemiology University of Saskatchewan Saskatoon, SK, Canada Email: sya277@mail.usask.ca *Paul Hackett* SPHERU/Department of Geography and Planning University of Saskatchewan Saskatoon, SK, Canada *Roland Dyck* Canadian Center for Health and Safety in Agriculture Department of Medicine, College of Medicine University of Saskatchewan Saskatoon, SK, Canada
